# Miniaturized nanoelectrospray interface for coupling capillary electrophoresis with mass spectrometry detection

**DOI:** 10.1002/elps.202400090

**Published:** 2024-08-23

**Authors:** Tomáš Václavek, Elizaveta Vereshchagina, Leny Nazareno, Anand Summanwar, František Foret, Roman Řemínek

**Affiliations:** ^1^ Department of Bioanalytical Instrumentation Institute of Analytical Chemistry of the Czech Academy of Sciences Brno Czechia; ^2^ Department of Smart Sensors and Microsystems SINTEF Digital Oslo Norway

**Keywords:** capillary electrophoresis, electrospray ionization, mass spectrometry, microfabrication, proteomics

## Abstract

A miniaturized electrospray interface consisting of a microfluidic nanosprayer and nanospray module is reported in the presented short communication. The nanosprayer was fabricated using silicon (Si) technology suitable for cost‐efficient high‐volume mass production. The nanospray module enabled the positioning of the nanosprayer in front of a mass spectrometry entrance and its coupling with capillary electrophoresis based on the liquid junction principle. A case study of top‐down and bottom‐up proteomic analyses of intact cytochrome c and its tryptic digest demonstrates the practical applicability of the developed interface.

**Abbreviations**
HRSEM: high‐resolution scanning electron microscopy
SiRN: silicon‐rich silicon nitride

## 1 INTRODUCTION

Miniaturization represents an ongoing trend in analytical instrumentation due to the promise of higher analytical efficiency, increased sample throughput, and significantly lower cost per analysis compared to conventional systems. Capillary electrophoresis (CE) answers these demands well, as micrometer‐scale dimensions of the fused‐silica capillary used as a separation column enable highly efficient analyses with minimal sample and other chemicals consumption. Furthermore, since its introduction in the late 1980s, CE coupled with mass spectrometry (MS) detection has developed into one of the most versatile and powerful analytical tools that was applied for studies of analytes ranging from simple ions to whole cells [1, 2, 3]. ESI is the most convenient approach for electromigration‐based separation techniques and, therefore, represents the major choice for online CE–MS coupling [4]. The sample components are separated in the liquid phase and by ESI they are transferred into a gas phase under atmospheric pressure. This transfer starts with the formation of highly charged droplets that, under evaporation, create gas‐phase ions for MS analysis. Since ESI standardly produces multiply charged ions with minimal fragmentation, its utilization substantially extends the mass range of an MS system and is thus suitable for analyses of even intact (bio)macromolecules, such as in top‐down proteomics [5]. The sheath flow and sheathless interfaces are the two main ESI arrangements. In addition, an idea to combine the strengths of both these approaches has led to the introduction of a liquid junction concept [6]. In this setup, the separation capillary and ESI emitter are electrically connected via a small gap permanently filled with a spraying liquid. Decoupling the CE and ESI processes allows their independent optimization similar to the sheath liquid interfaces; however, the sample dilution is significantly limited maintaining sensitivity comparable with sheathless systems. For these reasons, a miniaturized ESI interface for CE coupling with MS detection was developed, and a preliminary application study was carried out in the presented work. Although other microfabricated ESI interfaces based on Si technology have already been published [7], the system reported here is, to the best of our knowledge, the first microfabricated system enabling direct coupling with CE using the principle of liquid junction.

## 2 NANOESI INTERFACE

This interface consists of a nanosprayer and a nanospray module. The nanosprayer is a microfluidic device designed for electrospraying. A nanospray module provides a miniaturized housing for the nanosprayer and allows both coupling to CE and operating it in front of an MS system entrance.

**FIGURE 1:**
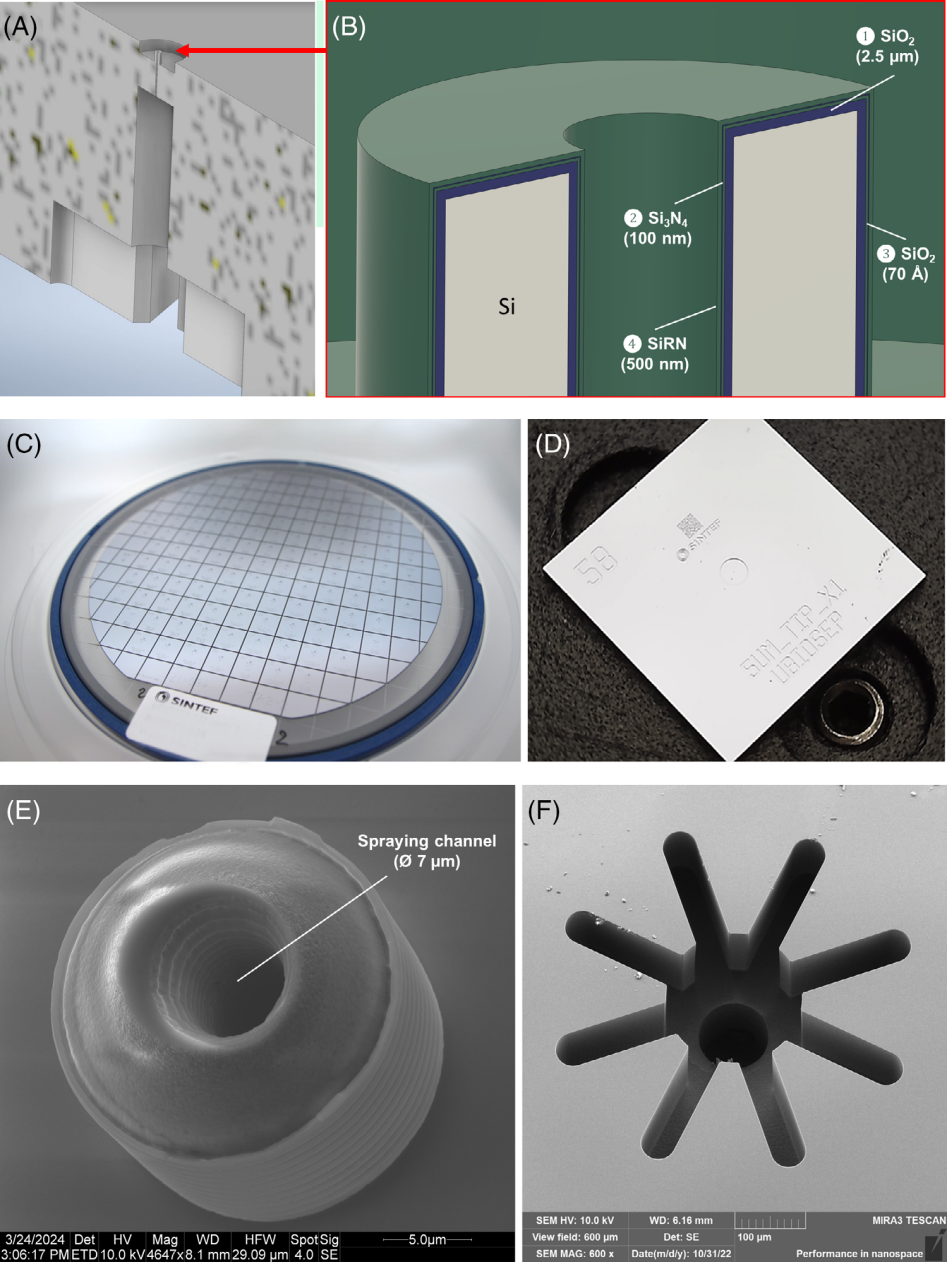
The microfabricated nanosprayer and its main features: (A) the scheme depicting the cross‐section of the nanosprayer; (B) the detailed scheme of the emitter tip with marked dielectric layers; (C) a processed wafer after dicing; (D) a single die, ca. 10 mm × 10 mm; (E) the high‐resolution scanning electron microscopy (HRSEM) images of the emitter tip with 7 µm spraying channel; and (F) the star‐shaped, self‐aligning liquid junction structure on the back side of the nanosprayer.

The nanosprayer and its main features are depicted in Figure 1. It incorporates an emitter tip on the front side of the Si wafer with a spraying channel in the middle that extends across the substrate toward a star‐shaped liquid‐junction structure on the opposite side of the chip (see Figure 1A). This structure was previously introduced elsewhere [8]; however, in that case, the microfluidic spraying chip was made of polyimide and featured a longitudinally positioned spraying channel, which was prone to clogging. The nominal inner diameter of the emitter, which is equal to the diameter of the spraying channel, is 7 µm. These dimensions enable operating the interface at very low flow rates, on the order of 100 nL/min and less, providing a high ionization efficiency and sensitivity [9]. The outer diameter of the emitter is 21 µm, leaving the emitter wall approximately 7 µm thick. The emitter tip is 15 µm tall. The nanosprayers were microfabricated using n‐type, double‐side polished, 150 mm diameter, and 400 µm thick Si wafers. Both the front and back sides of the wafer were patterned using a combination of UV‐lithography, reactive‐ion etching, and dry reactive‐ion etching techniques to complete the nanosprayer. The developed etching processes allow for the etching of high aspect ratio cavities in Si, in this case, especially concerning the spraying channel within the emitter, uniform in diameter. White light interferometry, high‐resolution scanning electron microscopy, and optical microscopy were used to assess the microstructures during processing. Further design details and overview of the developed production process were previously published elsewhere [10].

In principle, a dielectric layer formed on the Si substrate surface is essential to electrically isolate the voltage in the sprayed liquid from the conductive Si substrate. This layer consisted of a stack of four dielectric thin films: SiO_2_ (2.5 µm), Si_3_N_4_ (100 nm), SiO_2_ (70 Å), and SiRN (500 nm). A 3D cross‐sectional view is shown in Figure 1B. Interestingly, the semi‐conductive Si substrate can be also used as an additional counter electrode [11]. To do so, the entire dielectric stack was removed on the outer surface of the nanosprayer, and a thin‐film metal top surface electrode was patterned to provide a conducive connection to the Si substrate. The surface electrode was formed by sequential sputtering of an 80 nm chromium layer and a 60 nm layer of gold. Although gold provides excellent electrical contact, the chromium mid‐layer ensures stable adhesion to the Si substrate.

For this reason, the nanospray system requires two power supplies and two related electrical circuits for its operation. The first power supply, in our case the CE 7100 system (Agilent), provided high voltage via a stainless steel electrode inserted directly in the liquid reservoir with BGE (CE inlet). The voltage in this circuit was grounded via another stainless‐steel electrode placed in the sheath‐liquid reservoir (CE outlet), and it was dedicated for driving either the CE separation or electrospray ionization. This circuit was controlled by Agilent ChemStation software. The second power supply applied voltage on the nanosprayer Si substrate via the surface electrode. Applying voltage on the Si substrate modulated the electric field and its distribution around the emitter, which, as a consequence, significantly decreased the voltage threshold for electrospray onset and operation. The shape of the electrospray plume and stream of ions flying toward the MS entrance, which served as the counter electrode, could be controlled by tuning the voltage applied to the Si substrate. Additionally, a 22 kΩ resistor was connected in series in the secondary circuit and a voltage difference across this resistor was monitored by the Clarity chromatography station (Data Apex) in order to measure the leakage current. The leakage current measurement served for the assessment of dielectric layer breakdown because when the dielectric layer broke down, the nanosprayer was no longer able to electrospray and was replaced. Functional testing of the nanosprayer showed a stable performance and high ionization efficiency at flow rates below 100 nL/min [10].

**FIGURE 2:**
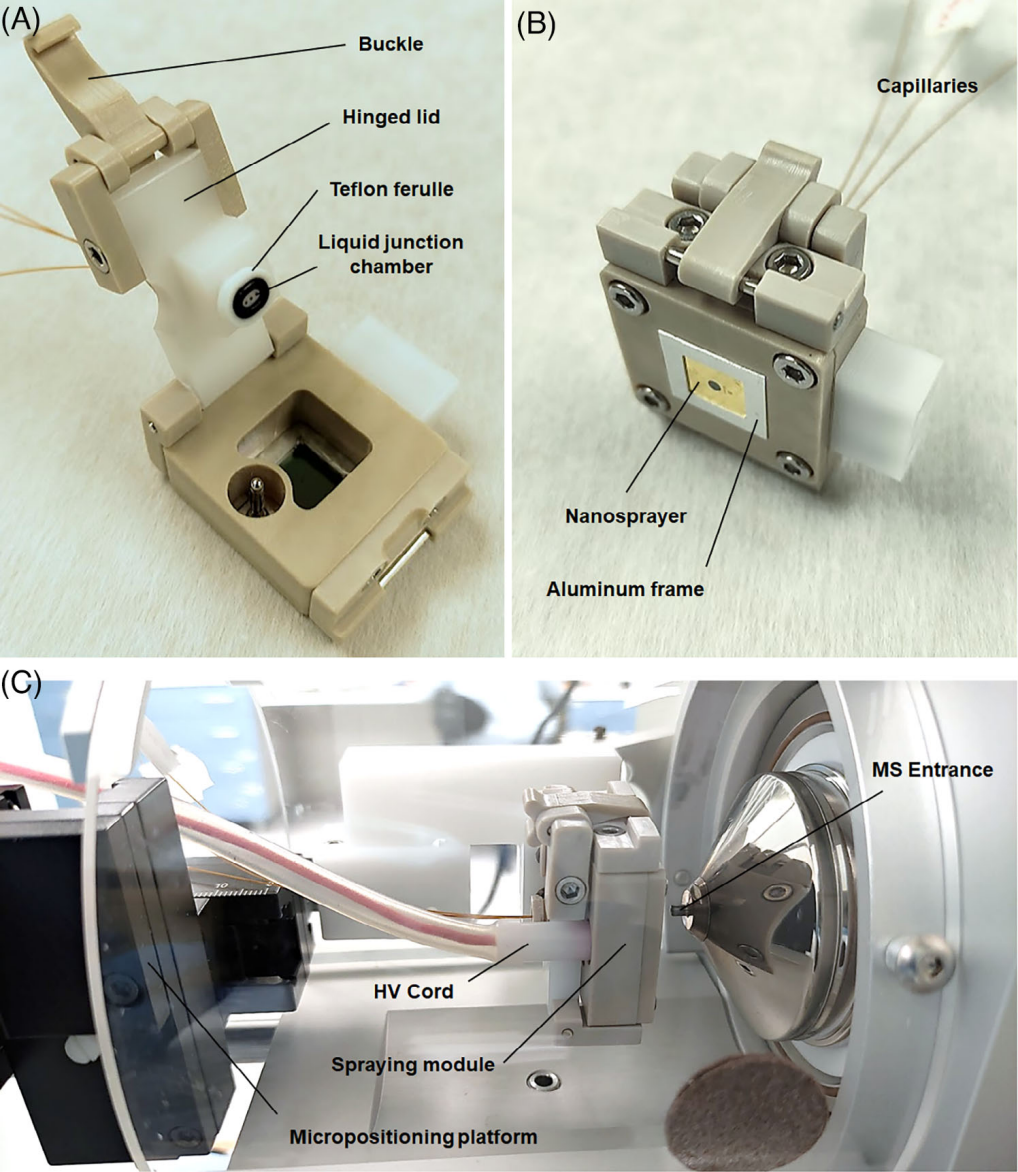
The photographs of the nanospray module: (A) the backside view of the module in the open position; (B) the frontside view of the module with installed nanosprayer; and (C) the installation of the interface to Thermo Fisher Scientific Velos Pro Dual‐Pressure Linear Ion Trap mass spectrometry (MS) system.

The nanospray module, designed as a rigid housing for nanosprayers, is depicted in Figure 2. The nanosprayer is positioned into an aluminum frame (see Figure 2B), enabling the voltage application on its substrate from an external HV power source. The module integrates three capillaries and an O‐ring in a custom‐designed Teflon ferrule that defines the volume of a liquid‐junction chamber. In this arrangement, the separation capillary from the CE system is positioned in the center to align with the star‐shaped structure at the back side of the nanosprayer, after the liquid‐junction chamber is tightened by pressing the hinged lid against the nanosprayer. The buckle system operating the hinged lid enables a quick and straightforward (dis)assembly or chip exchange. The other two capillaries, an auxiliary capillary delivering the spraying liquid and the waste capillary serving to drain the liquid from the chamber to a waste container, are positioned longitudinally on both sides of the separation capillary with a spacing of 800 µm (center to center). The system thus allows for automated flushing of the liquid‐junction chamber after each analytical run. The body of the module, hinge adapter, and buckle system were fabricated by CNC milling from PEEK plastic, providing excellent mechanical and chemical resistance properties that are retained at high temperatures. The hinged lid incorporating the Teflon liquid‐junction ferrule is made of POM‐H plastic. The overall dimensions of the nanospray module were 42 mm × 32 mm × 22 mm, excluding capillaries and HV cord. The dimensions are an important factor facilitating the integration of this module into various MS ion source platforms. Additional images of the nanospray module and the nanospray interface assemblies, and the installation arrangement of the nanospray interface and CE and MS systems can be found in the Supporting information section (Figures S1–S3).

## 3 APPLICATION STUDY

**FIGURE 3:**
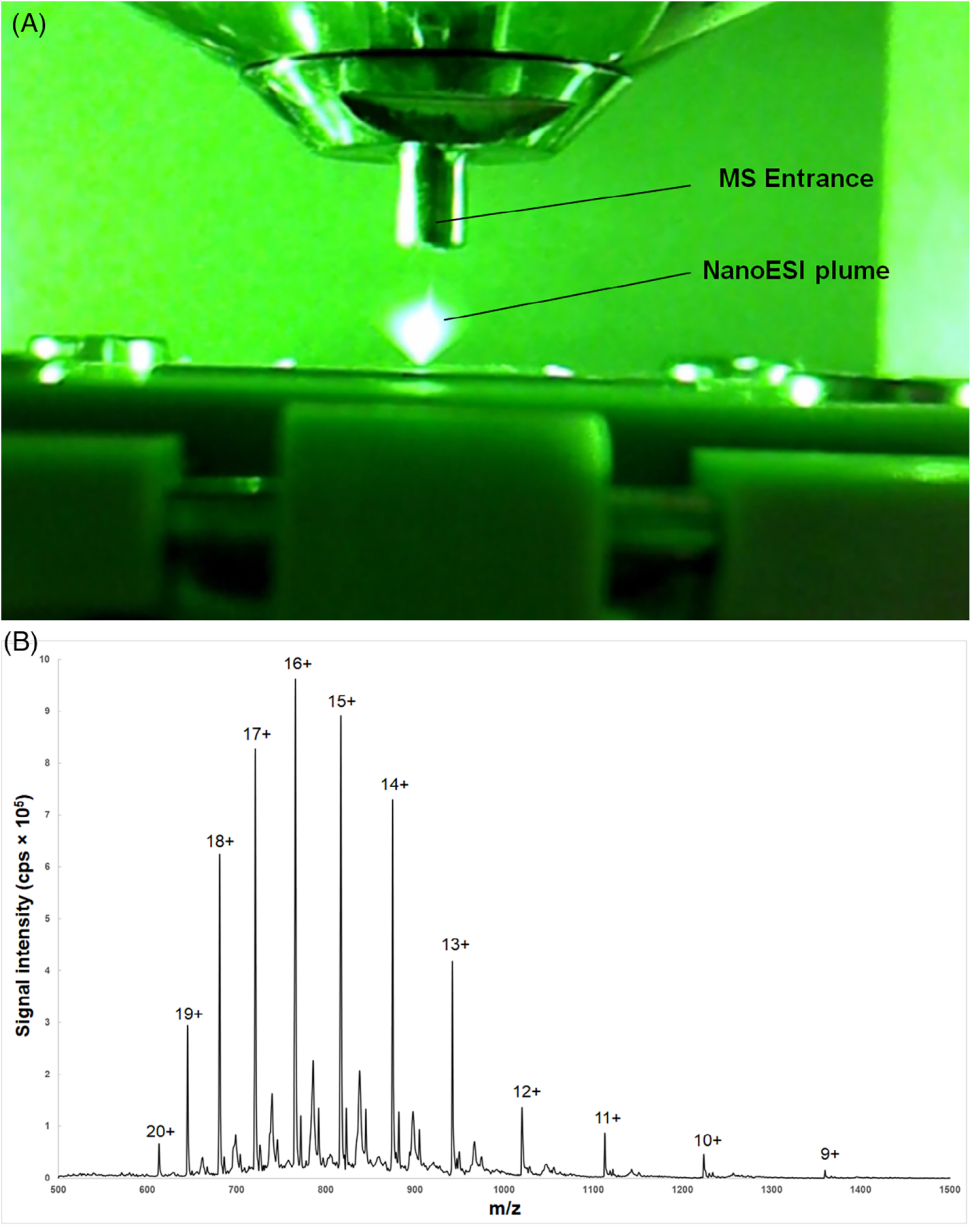
The analysis of intact cytochrome c: (A) the example of the interface performance when coupled with the mass spectrometry (MS) system—the spraying plume was illuminated with a green 532 nm laser; (B) the typical mass spectrum of multiply‐charged cytochrome c ions produced by nanospray obtained at full scan mode, the charge values were determined using the ESIprot Online software. The experimental conditions are summarized in Table S1 in the Supporting Information section.

The practical applicability of the miniaturized electrospray interface was demonstrated using two application cases. First, analysis of intact cytochrome c was performed using a CE‐nanoESI/MS method and continual injection of the sample. Cytochrome c is a small and highly soluble heme‐containing protein, playing one of the key roles in cell apoptosis. For this reason, it represents an efficient biomarker of various diseases, for example, non‐small cell lung cancer, drug‐induced liver injury, myocardial infarction, or drug‐induced acute kidney injury [12]. The analyses were carried out using the Velos Pro Dual‐Pressure Linear Ion Trap MS system (Thermo Fisher Scientific) and the 7100 CE system (Agilent Technologies). A 0.1 mg/mL standard of cytochrome c from bovine heart (Sigma Aldrich) prepared in 50 mM acetic acid used as BGE was continuously delivered to the nanospray interface using a bare fused‐silica (separation) capillary (Polymicro Technologies) with an inner diameter of 25 µm and outer diameter of 375 µm. A 50% v/v methanol prepared in BGE was used as a spraying liquid. The MS instrument was operated in positive ion mode and a voltage of 4.2 kV (positive polarity) was applied to the spray liquid reservoir and 200–450 V (positive polarity) to the Si substrate of the nanosprayer. After applying 0.8 bar in the system, the nanosprayer delivered the sample at a flow rate of approximately 85 nL/min. The CE‐nanoESI/MS system configuration and the other method conditions are discussed in detail in Sections S1.1–3. The plume observed under these conditions using the microscope camera is shown in Figure 3A. A typical spectra record observed at full scan mode (*m*/*z* = 500–1600) showing a characteristic envelope of cytochrome c is depicted in Figure 3B. Although the acidic conditions are necessary for cytochrome c unfolding, as the protonation of His18 coordinating to the heme iron plays a key role in this process [13], a significant effect of methanol presence was also noted. Only two cytochrome c ions were detected before methanol concentration in BGE was increased from 25% to 50% (data not shown). The developed system achieved a signal‐to‐noise ratio of approximately 230:1 with an infusion of 8.3 nM cytochrome c solution. This value is lower than the 450:1 reported in a previous study using a 10 nM solution [7]. Nevertheless, the sensitivity of both systems is likely comparable. This difference is attributed to the liquid junction within our system, which causes partial dilution of the sample.

**FIGURE 4:**
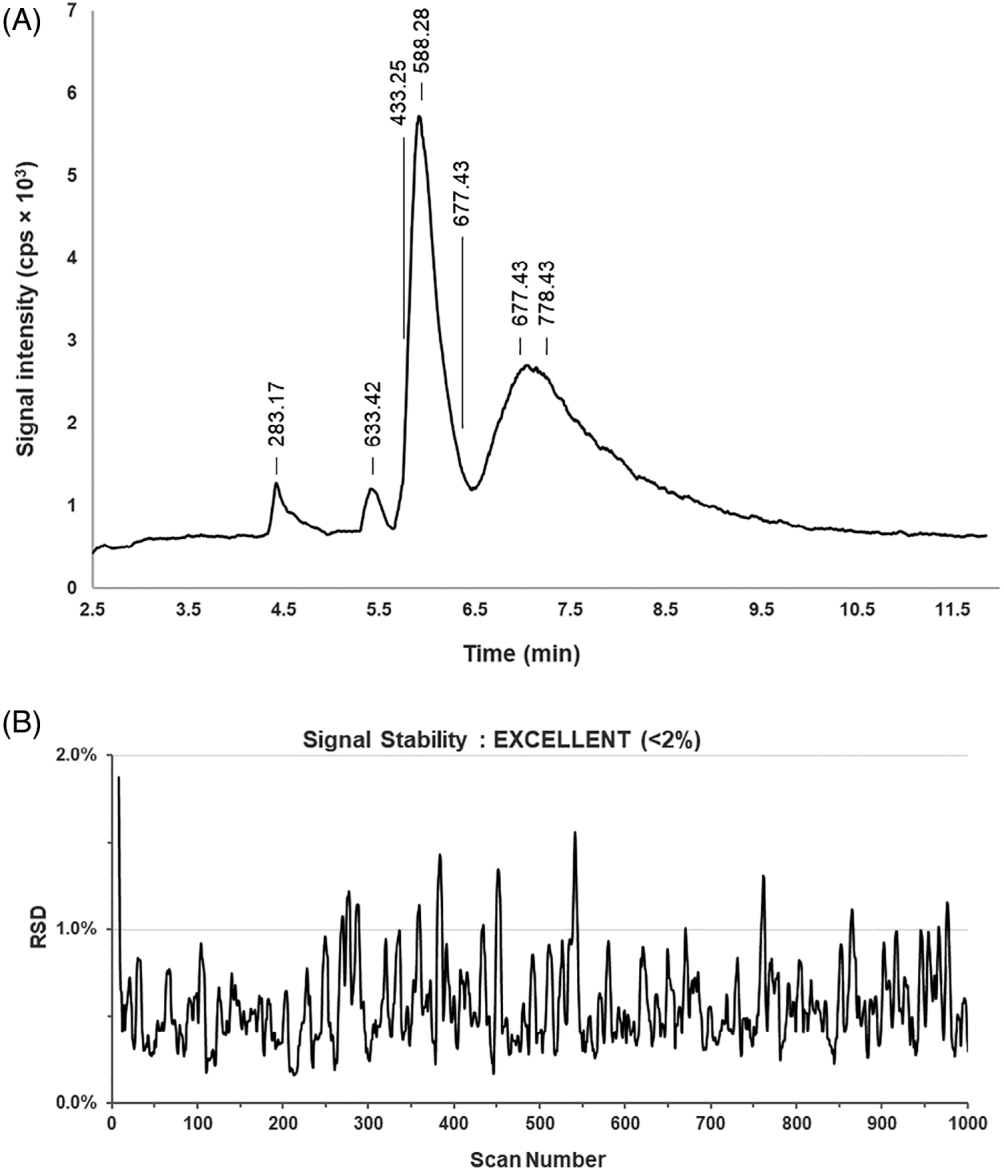
The analysis of the tryptic digest of cytochrome c: (A) the base peak electropherogram of the tryptic digest of cytochrome c (17 µg/mL), specific fragments are labeled by their masses: HK (*m*/*z* = 283.17), ATNE (*m*/*z* = 433.25), GDVEK—acetylated (*m*/*z* = 588.28), IFVQK (*m*/*z* = 633.42), YIPGTK (*m*/*z* = 677.43), and MIFAGIK (*m*/*z* = 778.43); (B) the record of the signal stability evaluated using the Tune Plus diagnostic software tool. The experimental conditions are summarized in Table S2 in the Supporting Information section.

Second, the separation and detection of a peptide mixture obtained by tryptic digestion of cytochrome c used as a model sample typical for the bottom–up proteomic studies were carried out. The sample was separated by application of 15 kV (positive polarity) for 5 min, and the separated zones were subsequently electrosprayed to the MS system by concomitant application of 2.2 kV and 0.8 bar. The sample preparation procedure and other optimized method conditions are summarized in Section S1.4. A typical base peak electropherogram of cytochrome c tryptic digest is shown in Figure 4A. Six specific fragments of cytochrome c were detected. The nanospray stability was also evaluated using Tune Plus diagnostic software (Thermo Fisher Scientific). This software assessed signal stability by calculating RSD values of the base peak intensity over the last 10 scans. The results, depicted in Figure 4B, demonstrate excellent spraying stability for the developed ESI interface. The promising performance of the presented system thus shall pave the way for further development of microfluidic platforms for bioanalytical applications.

## CONFLICT OF INTEREST STATEMENT

The authors declare no conflicts of interest.

## Supporting information

Supporting‐Information

## Data Availability

The data that support the findings of this study are available from the corresponding author upon reasonable request.
