# Supplementary material for: Miniaturized nanoelectrospray interface for coupling capillary electrophoresis with mass spectrometry detection
Source: Electrophoresis. 2024 Aug 23;45(21-22):1988–94. doi: 10.1002/elps.202400090 (PMC11662199; doi:10.1002/elps.202400090)
Supplement: Supplementary file 1 — Supporting‐Information [file ELPS-45--s001.pdf]

Supporting Information

Miniaturized nanoelectrospray interface for coupling capillary electrophoresis with mass spectrometry detection

Tomáš Václavek,[1] Elizaveta Vereshchagina,[2] Leny Nazareno,[2] Anand Summanwar,[2] František Foret,[1] Roman Řemínek[1*]

1 Department of Bioanalytical Instrumentation, Institute of Analytical Chemistry of the Czech Academy of Sciences, v. v. i., Veveří 967/97, 636 00 Brno, Czechia

2 Department of Smart Sensors and Microsystems, SINTEF Digital, Gaustadalléen 23C, 0373 Oslo, Norway

*Correspondence should be addressed to the following author(s):

Dr. Roman Řemínek
Department of Bioanalytical Instrumentation
Institute of Analytical Chemistry of the Czech Academy of Sciences, v. v. i.
Veveří 967/97, 636 00 Brno, Czechia
reminek@iach.cz

S1 Experimental Conditions

S1.1 Chemicals and Reagents

Ammonium acetate, ammonium hydroxide, cytochrome c from bovine heart, trypsin from porcine pancreas, and diclofenac were purchased from Sigma-Aldrich (Steinheim, Germany). Acetic acid was from Lach-Ner (Neratovice, Czech Republic). Methanol (MeOH) was supplied by Merck (Darmstadt, Germany) and sodium hydroxide by Penta (Praha, Czech Republic). All chemicals used were of analytical grade purity or the best purity available. All aqueous solutions were prepared using deionized water from a Purite Neptune Ultimate system (Thame, UK).

S1.2 CE-nanoESI/MS System

All the analyses were carried out using Velos Pro Dual-Pressure Linear Ion Trap MS system (Thermo Fisher Scientific, USA) and 7100 CE system (Agilent Technologies, USA). The nanoESI interface consisted of a nanosprayer and a nanospray module. The nanosprayer is a microfluidic device designed for electrospraying. A nanospray module provides a miniaturized housing for the nanosprayer and allows both coupling to CE and operating it in front of an MS system entrance. The assemblies of the nanospray module and the nanoESI interface are described in **Figures S1** and **S2**. The installation arrangement of the interface and CE and MS systems is depicted in **Figure S3**.

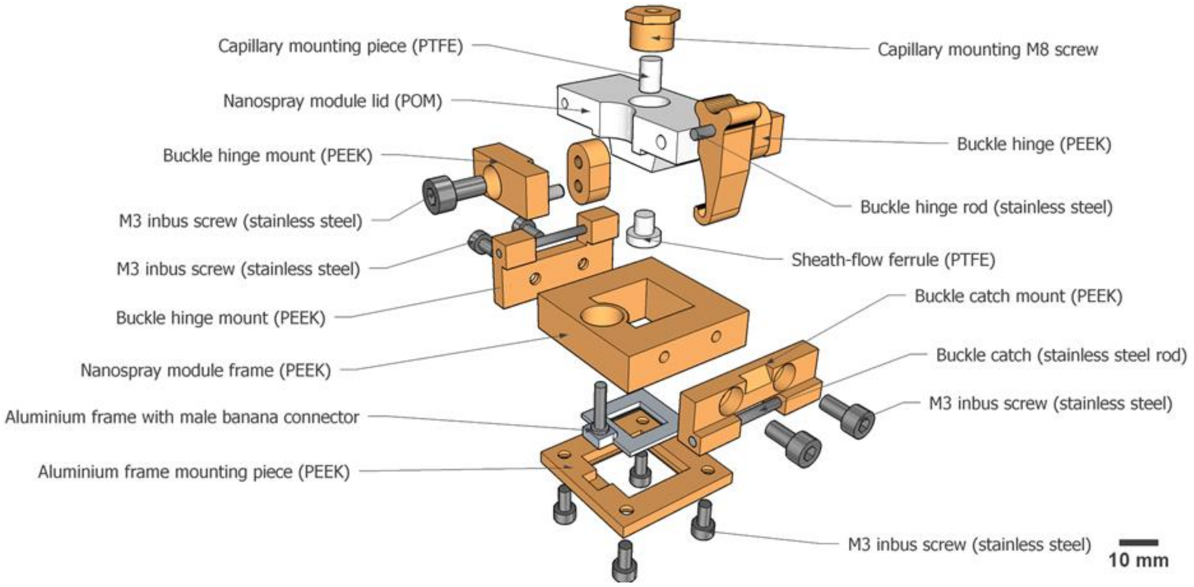

Figure S1: The exploded 3D model of the nanospray module.

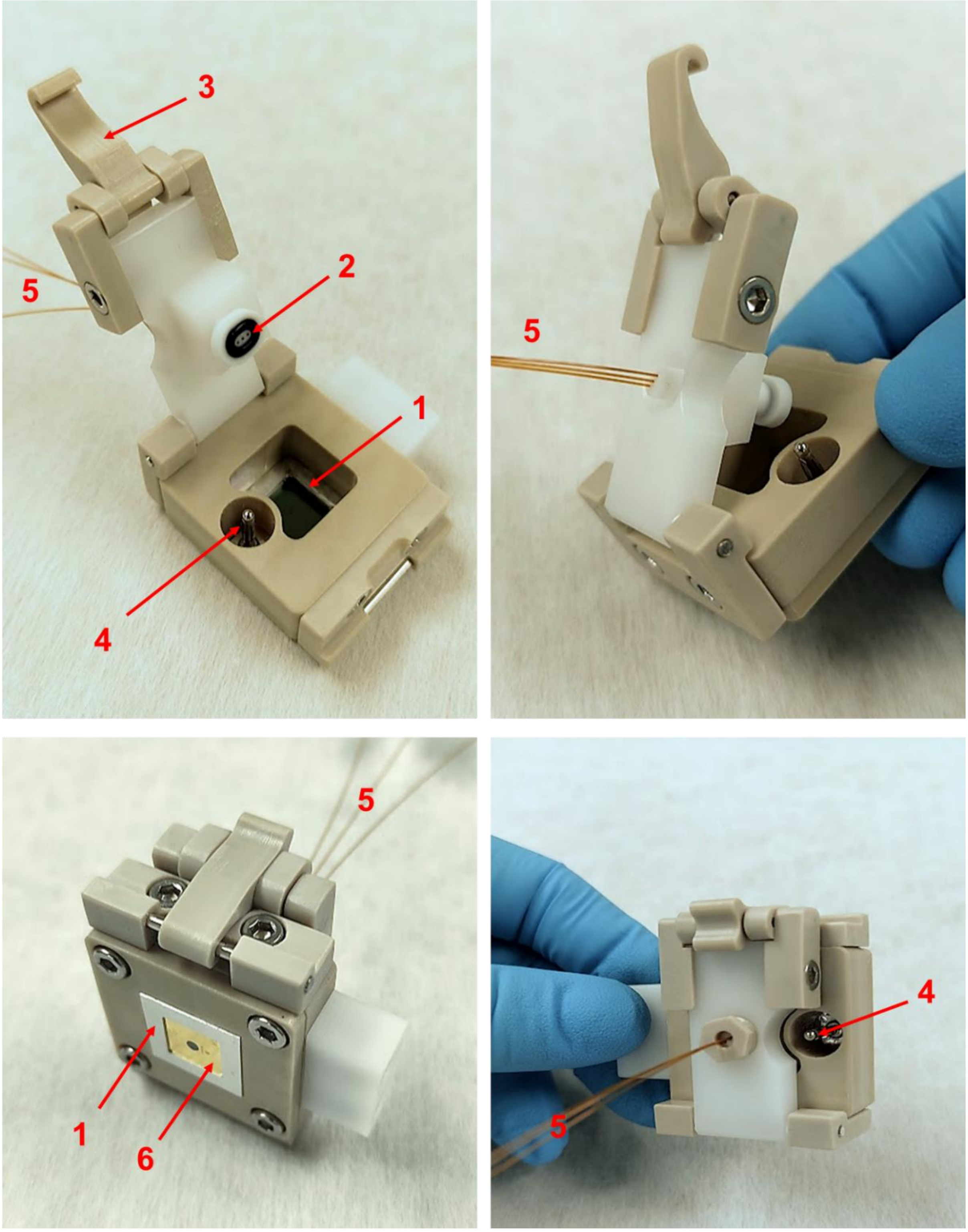

Figure S2: The assembly of the nanospray module depicting the aluminum frame used for housing the nanosprayer (1), o-ring defining the liquid junction (2), the buckle used for tightening the module (3), the integrated male banana connector for application of voltage on the aluminum frame and thus the Si substrate of the nanosprayer (4), the capillaries, the separation capillary is in the middle position to

align with the liquid junction structure on the back side of the nanosprayer (5), and the nanosprayer (6).

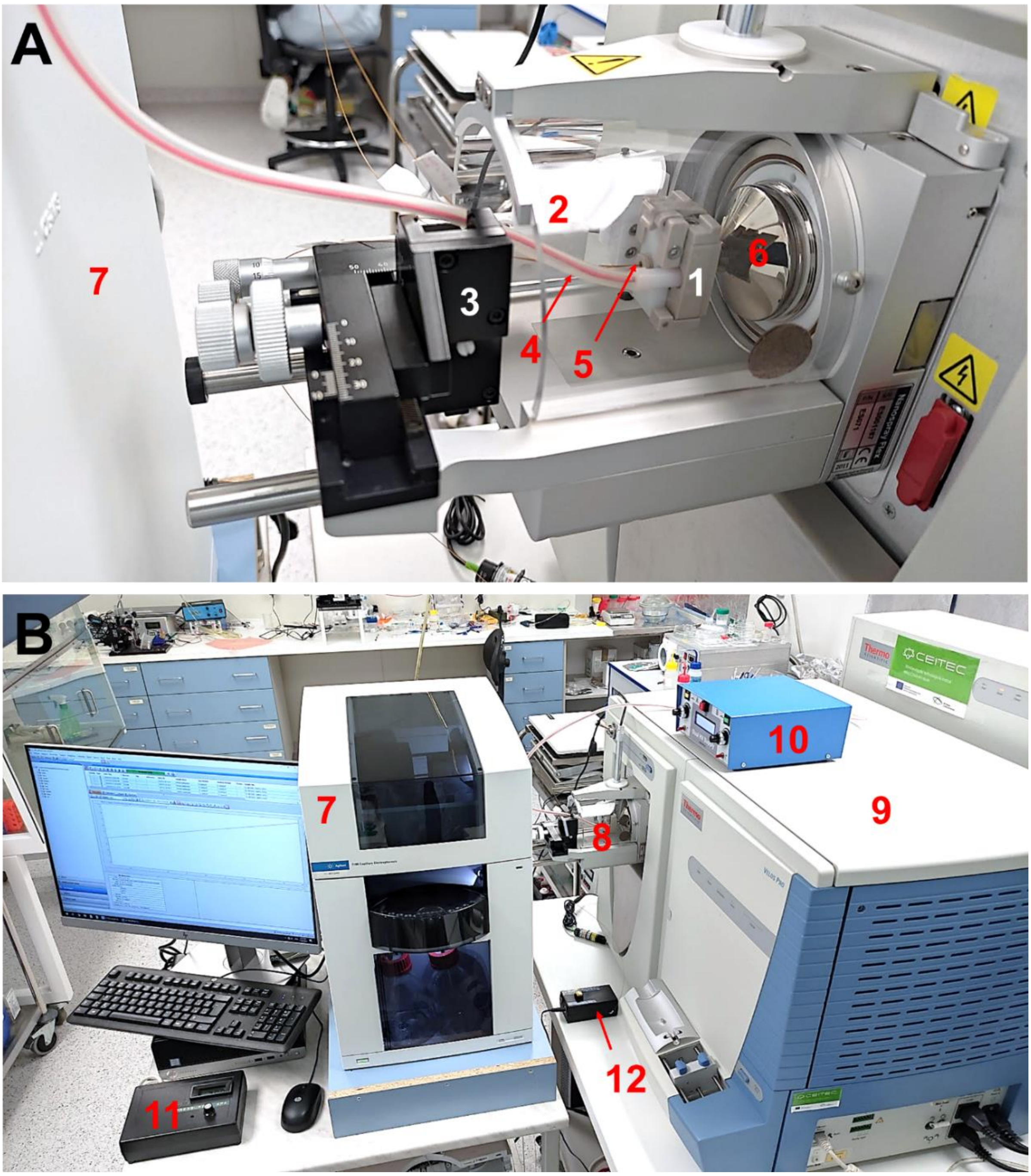

Figure S3: The CE-nanoESI/MS components. (A) The installation of the nanoESI interface into the Thermo Fisher Scientific Velos Pro Dual-Pressure Linear Ion Trap MS system; (B) the instrumental arrangement. Numbers denote the nanospray module (1), the quick-mount magnetic adapter (2), the 3-axis micropositioning platform (3), the wire connecting the nanosprayer with external HV power supply (4), the capillaries (5),

the MS entrance (6), the CE system (7), the interface (8), the MS system (9), the external HV power supply (10), the external gas pressure regulator (11), and the pinch valve (12).

Nanosprayers having a single emitter tip and a spraying channel with an inner diameter of 7 μm, and with deposited dielectric layers of SiO2 (2.5 μm), Si_3N_4 (100 nm), SiO_2 (70 Å), and SiRN (500 nm) were used for all the analyses. A sample was separated and/or delivered to the nanospray interface using a bare fused-silica (separation) capillary (Polymicro Technologies, USA) with an inner diameter of 25 μm, outer diameter of 375 μm, and total length of 60 cm. A bare fused silica capillary with an inner diameter of 50 μm, outer diameter of 375 μm, and total length of 70 cm was used as the auxiliary capillary, delivering spraying liquid into the system. The CE cassette was thermostated at 25°C. A bare fused silica capillary with an inner diameter of 150 μm, outer diameter of 375 μm, and total length of 80 cm was used as the waste capillary used for flushing the interface between the runs. During the analyses, the waste capillary was sealed by a pinch valve. Before its first use, the separation capillary was activated by rinsing with 1M NaOH for 30min, 0.1M NaOH for 10 min, and deionized water for 10 min at 50 °C. This procedure was accomplished at a pressure of 1.5 bar (150 kPa). Data was collected by a personal computer equipped with LTQ Tune Plus (Thermo Fisher Scientific, Waltham, MA, USA) software, and the results obtained were evaluated using Xcalibur™ (Thermo Fisher Scientific, Waltham, MA, USA) and MS Excel (Microsoft, Redmont, WA, USA) software.

The method conditions specific to each application are discussed in the following sections.

S1.3 Application Case 1: Intact Cytochrome c

The first method enabling analyses of intact cytochrome c was conceptualized as the direct infusion, i.e., with a continuous sample injection during the run. The analyte was monitored in a full scan mode with the mass range of m/z = 500–1600.

A 1 mg/mL standard of cytochrome c from bovine heart was prepared in deionized water as a stock solution. The samples were obtained by diluting the stock solution with 50 mM acetic acid used as background electrolyte (BGE) to the final concentration of 0.1 mg/mL and kept at 4°C before use.

The optimized method conditions are summarized in **Table S1**.

Table S1: Method conditions for analyses of intact cytochrome c.

**CE**	
BGE	50 mM acetic acid
Spraying liquid	50% v/v methanol in BGE
Sample injection	Continuous – concomitant application of 4.2 kV (positive polarity) and pressure of 0.8 Bar
Voltage applied on nanosprayer	300 V (positive polarity)
Separation capillary rinsing	180 s with BGE after analysis
Auxiliary capillary rinsing	180 s with spraying liquid after analysis
**MS**	
MS ion mode	positive
Entrance capillary temperature	150°C
Detection	Full Scan mode
Mass range	m/z = 500–1600
Number of microscans	1
Maximal injection time	200 ms

S1.4 Application Case 2: Tryptic Digest of Cytochrome c

The second method enables separation and detection of the peptide mixture obtained by tryptic digestion of cytochrome c. The analytes were monitored in a full scan mode with the mass range of m/z = 130–1000.

Solutions of 0.02 mg/mL trypsin and 1 mg/mL cytochrome c were prepared in 50 mM ammonium acetate (pH 8.0) used as an incubation buffer. The enzymatic reaction was initiated by mixing the cytochrome c and trypsin at a protein-to-enzyme weight ratio of 50, and the assay was carried out in an Eppendorf Thermomixer (Hamburg, Germany) at a mixing frequency of 650 rpm and temperature of 37°C for 16 hours. The enzyme reaction was terminated by the addition of 20 µL glacial acetic acid (its resulting concentration in the mixture was 5 % v/v), and the incubation mixtures were frozen at

-20°C. Before analysis, all samples were thawed in warm water, 6-fold diluted with deionized water, and kept at 4°C before use.

The optimized CE-nanoESI/MS method conditions are summarized in **Table S2**.

Table S2: Method conditions for analyses of tryptic digest of cytochrome c.

**CE**	
BGE	50 mM acetic acid
Spraying liquid	50% v/v methanol in BGE
Sample injection	Application of 0.8 bar for 10 s
Separation voltage	15 kV (positive polarity)
Separation time	5 min
Spraying voltage and pressure	Concomitant application of 2.2 kV and 0.8 bar
Voltage applied on nanosprayer	350 V (positive polarity)
Separation capillary rinsing	300 s with BGE after analysis
Auxiliary capillary rinsing	300 s with spraying liquid after analysis
**MS**	
MS ion mode	positive
Entrance capillary temperature	150°C
Detection	Full scan mode
Mass range	m/z = 130–1000
Number of microscans	1
Maximal injection time	200 ms
